# A Multifactorial Regulation of Glutathione Metabolism behind Salt Tolerance in Rice

**DOI:** 10.3390/antiox11061114

**Published:** 2022-06-03

**Authors:** Sara Cimini, Vittoria Locato, Valentina Giacinti, Michela Molinari, Laura De Gara

**Affiliations:** Unit of Food Science and Nutrition, Department of Science and Technology for Humans and the Environment, Università Campus Bio-Medico di Roma, via Álvaro del Portillo 21, 00128 Rome, Italy; s.cimini@unicampus.it (S.C.); v.giacinti@unicampus.it (V.G.); michela.molinari@unicampus.it (M.M.); l.degara@unicampus.it (L.D.G.)

**Keywords:** abiotic stress, cell cycle, glutathione metabolism, hydrogen peroxide, miRNA395, *Oryza sativa*, rice, salt stress

## Abstract

Knowledge of the stress-induced metabolic alterations in tolerant and sensitive plants is pivotal for identifying interesting traits that improve plant resilience toward unfavorable environmental conditions. This represents a hot topic area of plant science, particularly for crops, due to its implication in food security. Two rice varieties showing dissimilar resistance to salt, Baldo and Vialone Nano, have been studied to investigate the mechanisms underpinning tolerance toward salinity, and these studies have focused on the root system. A detailed analysis of the salt stress-dependent modulation of the redox network is here presented. The different phenotype observed after salt exposure in the two rice varieties is coherent with a differential regulation of cell-cycle progression and cell-death patterns observed at root level. Baldo, the tolerant variety, already showed a highly responsive antioxidative capacity in control conditions. Consistently, stressed Baldo plants showed a different pattern of H_2_O_2_ accumulation compared to Vialone Nano. Moreover, glutathione metabolism was finely modulated at transcriptional, post-transcriptional, and post-translational levels in Baldo. These results contribute to highlight the role of ROS and antioxidative pathways as a part of a complex redox network activated in rice toward salt stress.

## 1. Introduction

Soil salinization is a major environmental challenge that reduces the yield of crops, which include several salt-sensitive species [1]. Indeed, an excessive soil-salt concentration reduces plant growth and productivity and can finally cause plant death. Soil salinization primarily depends on geological processes, such as those involving rock erosion and inland sea-water intrusion. Evolving climate conditions exacerbated this process by promoting drought and desertification, whereas anthropic activities stimulated salt accumulation in the soil, using poor-quality water for irrigation, deforestation, and changes to the use of land [2].

Even if different ions are responsible for an increase in soil salinity, the levels of Na^+^ and Cl^−^ are mainly taken into account, given that these ions are toxic for plants and since Na^+^ is particularly involved in the deterioration of the physical structure of the soil [3]. According to Food and Agriculture Organization of the United Nations (FAO) (1997), a soil is defined as saline when it has an electrical conductivity (EC) at least of 4 dS m^−1^ (equivalent to 40 mM NaCl).

The identification of molecular and genetic determinants of plant tolerance to salt is an important goal for identifying breeding and biotechnological strategies to increase crop resilience to salt. However, this is a complex matter, given that salt exposure is a multifaceted form of stress for plants. It firstly consists in osmotic and ionic stress and secondarily causes oxidative stress [4]. Osmotic stress depends on the fact that roots exposed to a soil containing an excessive salt concentration experience a reduced capability to absorb water. As a consequence of the reduced availability of soil water, plants undergo water deficit, and stomata closure promptly occurs. This event reduces CO_2_ supply and consequently carbon fixation by photosynthesis, causing plant growth inhibition. Therefore, salt-stressed plants generally show a modified ratio of roots to shoot dry biomass that favors the former [5].

By reducing transpiration, stomata closure also prevents long-distance transport of salts, preserving plant aerial parts from ionic stress. Na^+^ accumulation in shoots causes early leaf senescence, disrupting thylakoid membrane integrity and leading to chlorophyll degradation and thus it further compromises photosynthesis. Na^+^ accumulation in plant tissues causes various physiological disorders by promoting nutrient imbalance. High cytoplasmic concentrations of Na^+^ disrupts the uptake of other ions into plant cells. In particular, at high soil concentration, Na^+^ competes with K^+^, reducing the latter uptake of the latter and consequently leading to metabolic impairment. In fact, the maintenance of K^+^ homeostasis is crucial for the cells given that this ion is essential for cell turgor pressure, membrane potential, and enzymatic activities [4]. 

Ion imbalance and water deficit cause reactive oxygen species (ROS) over-production under salt conditions. Consequently, oxidative damage occurs within different cell compartments, further compromising structural and metabolic features [6]. ROS can also increase ion imbalance by activating guard-cell-outward-rectifying potassium channels (GORK) which mediate K^+^ efflux from the cells and thus causing K^+^ leakage [7].

Antioxidants can attenuate the damaging effects of ROS and increase plant tolerance, reducing cell damage in time. A fine modulation between ROS producing and scavenging systems plays a key role in determining the role of these reactive species as toxic molecules or crucial messengers involved in redox signaling [8,9,10]. In this context, the control of ROS levels by the up-regulation of antioxidant systems has often been correlated with salt tolerance by regulating several physiological responses [6]. In particular, high glutathione (GSH) levels have often been correlated with a higher salt tolerance [11], and sulfur supplementation has been found to increase salt-stress tolerance by increasing GSH levels and reducing salt-dependent oxidative stress [12,13]. However, the involvement of the GSH network in the defense response able to confer salt tolerance has been poorly investigated in rice.

Rice, a staple food for 50% of the world’s population, is also one of the most salt-sensitive crops among cereals. The study of intra-species variability has been considered an important tool for the identification of tolerant traits in rice. As an example, rice cultivars showing different sensitivity to salt were found differently able to manage redox impairment caused by this stress [14].

Previous studies identified Baldo and Vialone Nano as two Italian rice cultivars showing different susceptibility to salt stress [15]. In particular, the tolerant line, Baldo, showed a better capacity to maintain a K^+^/Na^+^ rate close to a physiological range within cells compared to the sensitive one, Vialone [16]. Baldo also mainly allocated Na^+^ in roots rather than in leaves, thus preserving photosynthesis [17]. At a cellular level, Baldo also showed a different salt-triggered hydrogen peroxide signature compared to Vialone Nano [16]. This suggests that Baldo cell cultures manage ROS levels better than Vialone Nano ones; these Baldo cell cultures thus promote an ROS signaling mode in order to activate defense responses against the stress [16]. Moreover, data obtained from experiments performed on Baldo and Vialone Nano plants suggested that GSH metabolism had a primary role in plant response to salt stress in these cultivars [17].

In this study, the different responses to high salinity of Baldo and Vialone Nano plants have been deeply investigated at root level through an analysis of phenotypic/morphological parameters and meristematic activity. The involvement of antioxidant systems in the activation of defense responses against salt stress was also reported. A major role in determining salt tolerance in Baldo was attributed to molecular mechanisms controlling GSH homeostasis under stressful conditions in this tolerant cultivar.

## 2. Materials and Methods

### 2.1. Plant Materials and Growth Conditions

*Oryza sativa* ssp. japonica seeds of two Italian varieties named Baldo and Vialone Nano were kindly provided by Bertone Sementi SPA (Terruggia, Italy) and by Consiglio Per La Ricerca In Agricoltura E L’Analisi Dell’Economia Agraria (CREA, Vercelli, Italy). Seeds were surface-sterilized for 1 min in 70% ethanol and rinsed four times with deionized water. Seeds were sown on water-wetted filter paper in glass Petri dishes and left to germinate for 3 days at 25 °C in the dark conditions. Seedlings were transferred in a hydroponic system containing a modified Hoagland solution [17,18]. Plants were grown until the vegetative stage V2 [19] at approximately 7 days in a growth chamber at 26/21 °C, with a 16/8 h photoperiod, RH of 70%, and light of 120–150 μmol photons m^−2^ s^−1^. Solutions were exchanged every 24 h to ensure that plants remained at a nutritional steady state in the hydroponic system.

Seedlings at the V2 stage were transferred in a new hydroponic solution with or without the saline solution (NaCl:MgSO_4_:CaCl_2_:NaNO_2_ = 10:2:1:1; with a NaCl concentration of 100 mM), treated and control condition respectively. Leaves and roots from control and treated plants were collected at different time points, washed with deionized water, frozen in liquid nitrogen, and stored at −80 °C for subsequent analyses.

### 2.2. Shoot and Root Phenotypic Characterization

#### 2.2.1. Determination of Plant Growth

The length and dry weight of shoots and roots were recorded for the measurement of plant growth. Shoot and root length was recorded from scanned images with Image J software. A minimum of 50 plants were considered for each time point.

The samples were dried at 70 °C for 3 days to determine the dry weight of both leaves and roots dry. 

#### 2.2.2. Leaf Color Quantification and Chlorophyll Content in Leaves

The color of the 2nd leaf of Baldo and Vialone Nano in control and treated conditions was recorded with the CIELab color space and converted to the RGB scale. In the CIELAB color coordinates system, L* represents the perceived lightness, and color coordinate a* and b* indicate the change from red to green and from yellow to blue, respectively [20]. The reported values are the means of four independent experiments. 

For the determination of chlorophyll content in leaves, samples were collected from fully developed leaves and their chlorophyll a and b (chl a and chl b) content was determined with the spectrophotometric method previously described [21] by Ferrer et al. (2018). Briefly, leaves were finely grinded in liquid nitrogen and homogenized with 100% cold methanol in a 1:50 ratio (*w/v*). The homogenate was centrifuged for 10 min at 20,000× *g* at 4 °C. The supernatants were used for the spectrophotometric determination of chlorophyll a and b content. Total chlorophyll a and b content was considered and normalized for g fresh weight (FW).

#### 2.2.3. Cell Root Viability

Root cell viability was determined by Evans Blue staining [22]. Root tips (3 cm) were incubated with a 0.25% (*w/v*) Evans Blue in aqueous solution for 30 min, then thoroughly rinsed with deionized water until no further blue dye was eluted from the roots. The stained root tips were observed under an optical microscope (DMLS, Leica, Wetzlar, Germany). To quantify the decrease in cell viability, the absorbed Evans blue stain was extracted by the roots. The roots were incubated in 1% (*w/v*) SDS dissolved in 50% (*v/v*) methanol at 50 °C for 30 min, and then the absorbance at 600 nm of the solution in which the roots were incubated was measured.

### 2.3. Gene Expression Analysis

Total RNA was extracted from rice roots with the TRIzol reagent (Invitrogen, Waltham, MA, USA), a procedure that followed the manufacturer’s instructions. Reverse transcription was performed from 1 μg of total RNA with poly (dT12–18) primer and reverse transcriptase (Applied Biosystems, Waltham, MA, USA). RT-qPCR was performed in a Light Cycler 7900HT FAST (Applied Biosystems) with SYBR^®^ Green. Primers were designed with Primer-BLAST (https://www.ncbi.nlm.nih.gov/tools/primer-blast/) (accessed on 1 September 2020) and are listed in Supplementary Table S1. Accumulation of mature miR395 was determined by stem-loop RT-qPCR [23]. Data were calculated using the 2^−^^△△Ct^ method [24,25]. The OsUBQ5 gene (Os01g0328400) and OsACT1 (Os03g0718100) were used as housekeeping genes. Four independent biological replicates with three technical replicates were analyzed.

### 2.4. Flow Cytometry

Samples were processed and nuclei isolated as described by Zhao et al. [26] with minor modification. For each sample, 10 of 0.5 cm segment root tips were collected. Root tips were fixed in 20 mL of fixative buffer (pH 7.5, 12.5 mM Tris, 12.5 mM Na_2_EDTA, 125 mM NaCl, 0.125% Triton X-100, 2% formaldeide) at 4 °C for 25 min. 

Fixed root tips were washed three times with a washing Tris Buffer (pH 7.5, 10 mM Tris, 10 mM Na_2_EDTA, 100 mM NaCl) and then homogenized at 4 °C in 1 mL of lysis buffer (pH 7.5, 14.9 mM Tris, 2 mM Na_2_EDTA, 20 mM NaCl, 80 mM KCl, 0.49 mM spermine tetrahydrochloride, 1% β-mercaptoethanol, 1 % Triton X-100). The homogenate was filtered through a 30 μm nylon filter (Millipore, Nylon Net Filters). A treatment with 2.5 μL of RNase 10 μg/μL for 10 min was performed at room temperature. Finally, treatment with PI (100 μg mL^−1^) was performed. Through the use of the flow cytometer (Beckman Coulter Life Sciences CytoFLEX benchtop flow cytometer), propidium iodide (PI) fluorescence intensity was determined with 488 nm excitation and 580 nm detection for a minimum of 30,000 nuclei per sample.

### 2.5. Analysis of Hydrogen Peroxide and Redox Systems 

H_2_O_2_ was measured according to Sabetta et al. [27]. Briefly, 0.3 g of roots from control and treated plants were harvested and quickly ground in liquid nitrogen and homogenized in 40 mM Tris-HCl pH 7.0 in presence of 20 μM of 2′,7′-dichlorofluorescein. The samples were incubated for 1 h in the dark. The H_2_O_2_ levels of the extracts were measured with a spectrofluorometer (excitation λ = 495 nm; emission λ = 530 nm) and normalized for g FW. 

Ascorbate (ASC) and glutathione (GSH) levels and relative redox states were measured as previously described in [28]. Root tips from control and treated plants were collected, ground in liquid nitrogen, and homogenized with 6 volumes of 5% meta- phosphoric acid at 4 °C. The homogenate was centrifuged at 20,000× *g* for 15 min at 4 °C, and the supernatant used for the analysis. 

The activities of ASC peroxidase (APX) (L-ASC:H_2_O_2_ oxidoreductase, EC 1.11.1.11), dehydroascorbate reductase (DHAR) (GSH: dehydroascorbate oxidoreductase, EC 1.8.5.1), GR (NADPH:GSH disulfide oxidoreductase, EC 1.6.4.2), monodehydroascorbate reductase (MDHAR; NADH: ASC free radical oxidoreductase, EC 1.6.5.4), and catalase (CAT; hydrogen-peroxide: hydrogen peroxide oxidoreductase, EC 1.11.1.6) were measured according to [29]. Root tips from control and treated plants were collected, ground in liquid nitrogen, and homogenized at 4 °C in 4 volumes of 50 mM Tris-HCl (pH 7.8), 0.05% (*w/v*) cysteine, 0.1% (*w/v*) BSA, and 1 mM ASC. The homogenate was centrifuged at 20,000× *g* for 15 min at 4 °C, and the supernatant was analyzed by spectrophotometry.

Lipid peroxidation was assessed by measuring the concentration of malondialdehyde (MDA) as the end product of lipid peroxidation process according to [30]. 0.1 g of root tips were homogenized with 1 mL of 50 mM phosphate buffer (pH 7.8) with a mortar and pestle and centrifuged at maximum speed for 15 min. Subsequently, 400 μL of supernatant were mixed with 1 mL of 0.5% thiobarbituric acid and incubated at 90 °C for 20 min. The mixture was then cooled and centrifuged, and the absorbance of the resulting supernatant was measured at 532, 600, and 450 nm. The MDA content was determined with the following equation: 6.45 × (A532—A600)—0.56 × A450.

Total antioxidant capability in control and treated roots was determined by the 2,2′-azino-bis(3-ethylbenzothiazoline-6-sulfonic acid (ABTS) assay and expressed as Trolox Equivalent Antioxidant Capability (TEAC) according to the method previously described by Pasqualetti et al. [31] with modifications. Root tips (approximately 0.1 g) from control and treated plants were collected, ground in liquid nitrogen, and homogenized with 2 mL 50 mM Na-phosphate buffer (pH 7.5). The homogenate was centrifuged at 10,000× *g* for 10 min at 4 °C and the aqueous phase was recovered. The pellet was resuspended in 10 mL of methanol and the mixture was centrifuged at 10,000× *g* for 10 min at 4 °C. The organic phase was separately collected. The antioxidant capability of the hydrophilic and lipophilic phases was measured. The radical cation ABTS^∙+^ was produced by incubating 7 mM ABTS buffer (5 mM NaH_2_PO_4_-H_2_O, 5 mM Na_2_HPO_4_-2H_2_O, pH 7.4) with 2.5 mM K_2_S_2_O_8_. The mixture was kept in the dark at room temperature for 16 h. The ABTS^∙+^ working solution was obtained by diluting 7 mM ABTS^∙+^ to a final absorbance of 0.70 ± 0.05 at 734 nm. A volume of 10 μL of diluted extract was mixed with 190 μL of ABTS^∙+^ working solution and the absorbance at 734 nm was monitored for 3 min. A calibration curve was prepared with Trolox as a standard used in a concentration range 0–700 μM. Results are expressed as μmol Trolox equivalent (TE) per mg of sample.

### 2.6. SDS/PAGE and Immunoblotting

For the analysis of the glutathionylation profile, proteins were extracted in a denaturation buffer containing 2.5% SDS, 10% glycerol, bromophenol blue, and 75 mM Tris-HCl (pH 6.8) at 100 °C for 10 min. Denatured proteins were then separated on 10% SDS-PAGE for 50 min at 200 V in the MES-Tris-SDS running buffer, and blotted to nitrocellulose membranes (Protean, Whatman^®^, Maidstone, UK) in the transfer buffer (25 mM Tris, 190 mM Gly, and 20% (*v/v*) methanol), at 100 V for 60 min, a procedure performed with a Bio-Rad Mini Trans-Blot cell. After transfer, the membrane was briefly rinsed in PBS-T (8 mM sodium phosphate, 2 mM potassium phosphate, 140 mM NaCl, 10 mM KCl, pH 7.4, 0.1%, *v/v* Tween 20) and then blocked with a solution containing 5% (*w/v*) skim milk powder, 150 mM NaCl, 20 mM Tris-HCl, pH 7.5, and 0.05% (*v/v*) Tween-20 for 90 min. The membrane was colored with Ponceau and probed with an anti-GSH monoclonal antibody. Primary antibody incubation was performed overnight at 4 °C with an anti-GSH monoclonal antibody (Dylite 549, Virogen, Watertown, MA, USA) 1:1000 dilution. Following primary antibody incubation, membranes were washed three times for 5 min each in PBST. Membranes were then incubated in an antimouse IgG-HRP conjugated secondary antibody (1:5000 dilution, Santa Cruz Biotechnology) for 1 h at room temperature. Bands were visualized by chemiluminescence (Clarity Western ECL, Biorad, Hercules, CA, USA) and the density of the blotting signals was quantified with the ChemiDoc MP and with the software ImageLab (BioRad, Hercules, CA, USA). The density of the blotting signals was normalized to Ponceau S [32].

### 2.7. Statistical Analysis 

All statistical analyses were performed with Prism 6 software (GraphPad Software, San Diego, CA, USA). One-way ANOVA and two-way ANOVA followed by Tukey test correction were performed for multiple comparisons, involving one or two independent variables, respectively. Statistical analysis Student’s t-test was applied when comparing only two sets of data. *p* < 0.01 and *p* < 0.05 were set as the significance cut-off. All values were presented as means ± SD.

## 3. Results

### 3.1. Morpho-Phenotypical Characterization of Baldo and Vialone Nano Plants Showed Different Salt Susceptibility 

**Figure 1:**
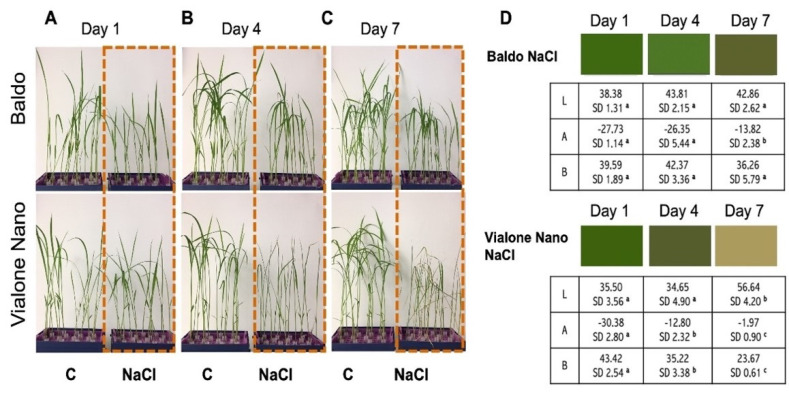
Effect of high salinity on rice seedlings. (**A**–**C**). Baldo and Vialone Nano plants in control and stress conditions. Seedlings were grown to the 2nd leaf stage (V2 stage) and then treated with 100 mM NaCl for 7 days. (**D**) Color of the 2nd leaf of Baldo and Vialone Nano in control and treated conditions recorded in the CIELab color space and converted to the red–blue–green scale. CIELAB expresses leaf color as three components: L component represents the perceptual lightness; A negative value was for green light component; A positive value was for red light component; B negative value was for blue light component; B positive value was for yellow light component. CIELab color values of the 2nd leaf were reported. The reported values are the means of four independent experiments ± standard deviation. Statistical significance was determined by one-way ANOVA followed by a Tukey test (*p* < 0.05). Different letters indicate significant difference. The statistical analysis was performed by comparing the values obtained for each light component (L/A/B) in leaves derived from NaCl plants over treatment time.

Baldo and Vialone Nano rice varieties were grown in hydroponics until V2 stage and then treated with 100 mM NaCl, which was reported as the best concentration able to highlight differences between Baldo and Vialone Nano without killing plants in the analyzed time [17]. A comparative phenotypic analysis showed significant differences between the two cultivars. Exposure to high salt concentration significantly perturbed morphological and physiological parameters of both genotypes; however, the salt treatment had a significantly greater impact on Vialone Nano than on Baldo (Figure 1A–D).

Indeed, a first phenotypic characterization revealed that Vialone Nano showed a progressive increase in the chlorotic areas on leaves starting from 4 days after salt treatment, whereas in Baldo suffering started to be evident only at seven days after salt treatment (Figure 1B,C). This was confirmed by an evaluation of leaf color, analyzed by CIELab color space, which revealed that Baldo treated plants were characterized by a higher green color component at 4 and 7 days after salt treatment in comparison with Vialone Nano treated plants (Figure 1D).

**Figure 2:**
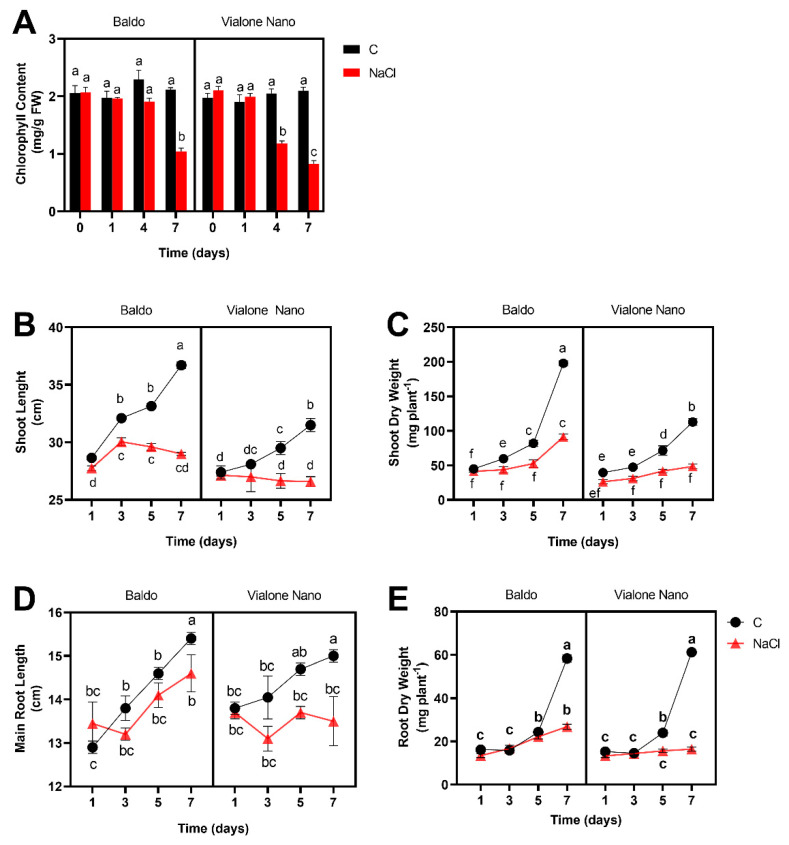
Morpho-Phenotypical Characterization of rice seedlings subjected to salt treatment. Baldo and Vialone Nano were grown under 100 mM NaCl for 7 days after salt treatment. (**A**) Leaf chlorophyll content; (**B**) Maximum shoot length; (**C**) Shoot dry weight; (**D**) Maximum root length; (**E**) Root dry weight. The reported values are the means of four independent experiments ± standard deviation. Statistical significance was determined by two-way ANOVA followed by a Tukey test (*p* < 0.01). Different letters indicate significant difference.

Accordingly, a stronger and more precocious reduction of chlorophyll content, a parameter related to the state of leaf chlorosis and photosynthetic efficiency, was more pronounced in Vialone Nano at 4 days after salt treatment than in Baldo. Treated Baldo plants showed an evident reduction only later, starting 7 days after salt treatment (Figure 2A). 

Consistent with these findings, the growth of shoots and roots was differentially compromised after salt treatment in Baldo and Vialone Nano. The analysis of the maximum shoot and root length as well as the relative dry weight underlined the higher susceptibility of Vialone Nano in comparison with Baldo. Salinity stress resulted in a significant reduction of shoot height, root length, and shoot and root DW in both varieties (Figure 2B–E). However, the growth of Vialone Nano was much more compromised, showing 34% and 10% reduction at 7 days after salt treatment in shoot and root length, respectively, in contrast with Baldo plants that present a reduction of 21% and 5%.

### 3.2. Salt Stress Mainly Compromised the Root Meristematic Activity and Root Cell Viability in the Sensitive Variety

To better understand the different outcome of growing showed by the two analyzed varieties, a closer look on the effects of salt stress on cell proliferation and mortality was conducted on the root apparatus. 

This was done because root growth and vitality prominently affect the healthy growth and yield of plants. Moreover, increasing evidence suggests that root development is profoundly involved in plant tolerance to abiotic stresses such as drought and salinity, thus influencing plant capability to overcome abiotic stress exposure [33,34,35].

**Figure 3:**
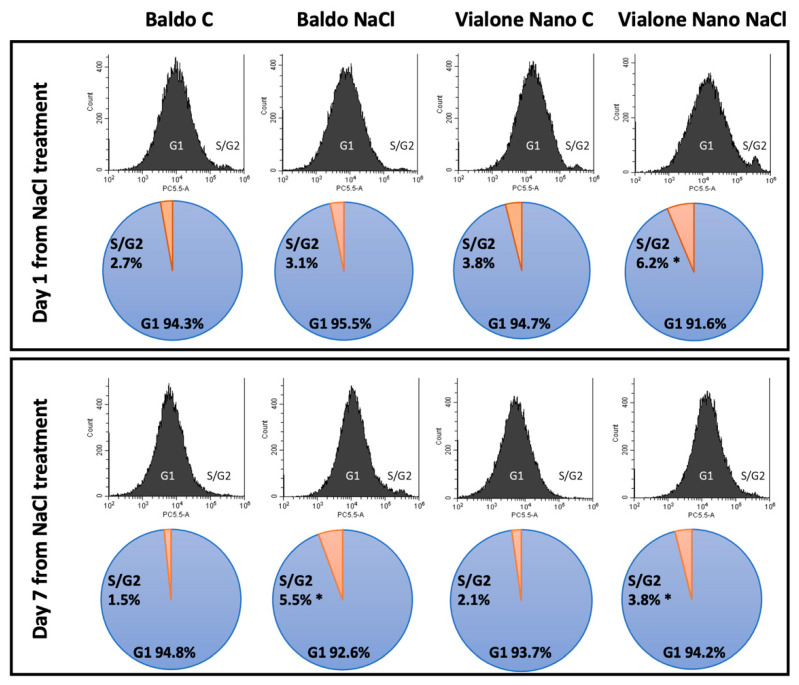
Cell cycle regulation in rice roots subjected to salt stress. Flow cytometric analysis of cell cycle of Baldo and Vialone Nano roots in control and salt stress conditions at 1 day and 7 days after salt treatment. All samples were stained with PI. Cell cycle progression in apical root meristem in response to salinity treatment. The analysis reflects the relative number of cells according to DNA content in each cell. The first peak (2n) represents the G1-phase and the second peak (4n) represents the S/G2 phase. Corresponding diagrams report the proportion of cells in G1 and S/G2 phases in Vialone Nano and Baldo under control and stress conditions. Data are presented as an average estimated from each treatment in five biological replicates with 30,000 events each. Student’s t-test was used to evaluate the significant variations between control and treated conditions for each variety. The reported values are the means of four independent experiments ± standard deviation. Statistically significant variations are shown (*) with *p*-value ≤ 0.05.

To understand the regulation of cell division and proliferation in the apical root meristem in response to salinity treatment, the cell cycle progression and the distribution between the phases of the cell cycle were examined by flow cytometry. This analysis revealed significant differences between Baldo and Vialone Nano starting from one day after stress imposition in the proportion of cells in each phase compared to control conditions. In Vialone Nano roots, nuclei in S/G2 phase (4C nuclei) began to accumulate early after salt exposure, passing from 3.8% to 6.2% after 1 day of salt treatment (Figure 3). This increase occurred at the expense of nuclei in G1 phase (2C nuclei), which account for 91.6% after salt stress compared to the 94.7% in the control conditions. In Baldo roots, this alteration of the cell cycle progression occurred later, starting from 7 days after salt treatment (Figure 3). In Baldo roots, nuclei began to accumulate in S/G2 phases passing from 1.5% to 5.5% at 7 days of treatment. These results indicate that exposure to salt stress conditions led to a rapid decrease in cell division rates in Vialone Nano causing DNA endo-duplication that preceded an early mitotic exit, which is generally a sign of the activation of death processes.

**Figure 4:**
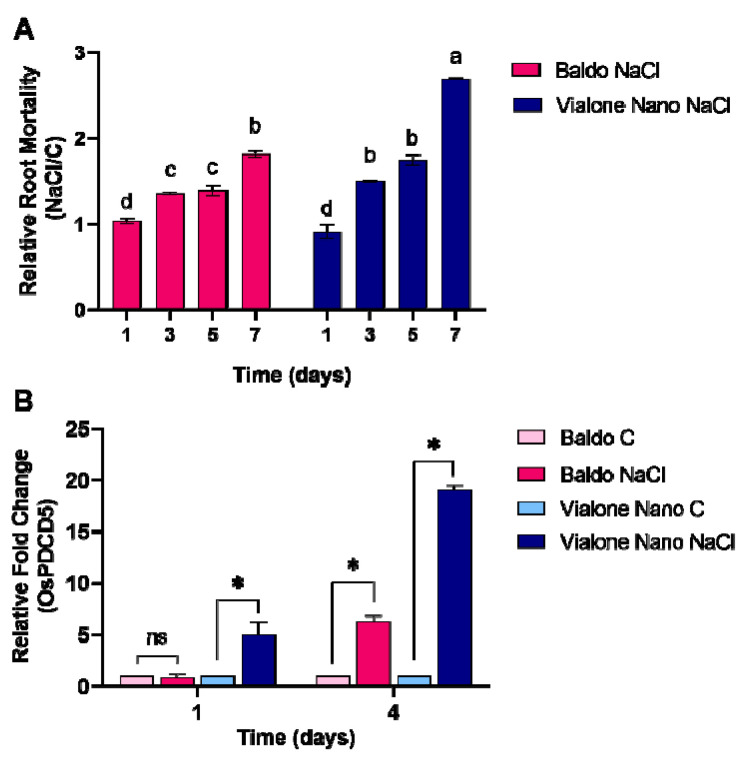
Cell death evaluation on salt-treated Baldo and Vialone Nano root apparatus. (**A**) Relative Root Mortality Ratio (NaCl/C plants) obtained from quantification of Evan’s Blue-stained root extract. (**B**) Expression of programmed cell death-related gene OsPDCD5 in control and treated plants. Data are presented as the mean standard deviation of at least three biological replicates. In (**A**) statistical significance determined by two-way ANOVA followed by a Tukey test (*p* < 0.01). Different letters indicate significant difference. In (**B**) Student’s t-test was used to evaluate the significant variations between control and treated conditions for each variety. Asterisks indicate significant differences between treatment and control by t-test with *p* < 0.01. ns, non-significant. The reported values are the means of four independent experiments ± standard deviation.

Accordingly, salt treatment affected the mortality of the root apparatus of the sensitive variety more rapidly and with a greater intensity compared to the tolerant variety. Three days after salt treatment, root mortality was increased by 25% and 40% in Baldo and Vialone Nano, respectively, and by 85% and 180% at 7 days over the corresponding control plants (Figure 4A).

In addition, the expression of programmed cell-death 5 (OsPDCD5), a gene known to be involved in programmed cell death (PCD) processes under different abiotic stresses such as cold temperature and UV-B irradiation [36,37,38], was examined one and four days after salt exposure in the root apparatus. OsPDCD5 presented a differential regulation of the gene expression that was upregulated both in Baldo and in Vialone Nano after salt stress but with a different timing and a different relative fold change. After one day of salt exposure, an increase in the relative fold change occurred specifically in Vialone Nano, while in Baldo this increase only occurred later (Figure 4B). 

### 3.3. The Tolerant Variety Showed Enhanced Capabilities to Activate the ROS Scavenging Systems That Enabled H_2_O_2_ to Act as a Signal Molecule

In response to salt stimuli, early signaling events in plants include modulation of ROS metabolism to maintain a delicate balance between ROS production and ROS-scavenging pathways [9,39]. ROS are small short-lived oxygen-containing molecules acting, when accumulated under a threshold concentration, as important modulators of defense and survival mechanisms and plant PCD. On the contrary, higher levels of ROS cause oxidative damage to cellular components such as lipids, DNA, and proteins, thus compromising intrinsic membrane properties essential for maintaining cell viability [40,41].

Therefore, an evaluation of the H_2_O_2_ accumulation and antioxidant metabolism in Baldo and Vialone Nano plants, both under control conditions and after salt exposure, was performed at a short time after stress application.

**Figure 5:**
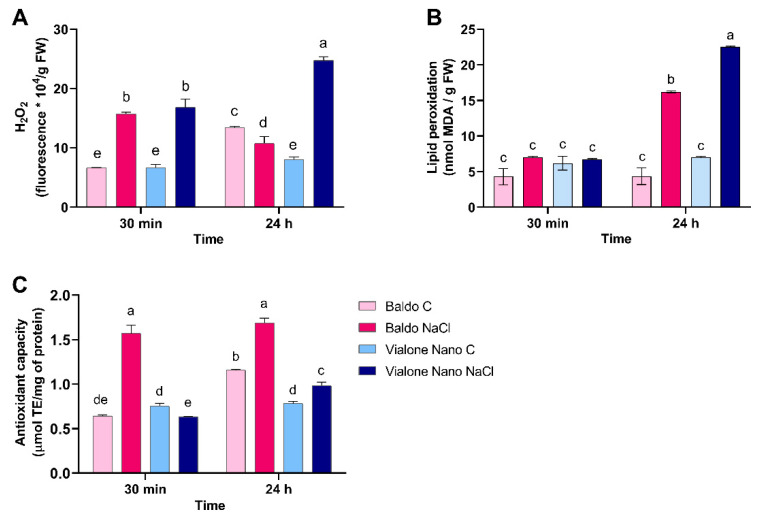
Redox markers in rice roots under salt stress. (**A**) Changes in H_2_O_2_ content; (**B**) levels of malondialdehyde (MDA) and (**C**) total antioxidant activity in Baldo and Vialone Nano roots subjected to salt stress. The reported values are the means of four independent experiments ± standard deviation. Statistical significance was determined by two-way ANOVA followed by a Tukey test (*p* < 0.01). Different letters indicate significant difference.

The quantification of the intracellular H_2_O_2_ amount was investigated in Baldo and Vialone Nano roots 30 min and 24 h after salt treatment (Figure 5A). H_2_O_2_ concentration increased at the same level in the two rice varieties at 30 min from stress exposure. At 24 h from the treatment, H_2_O_2_ levels continued to increase in Vialone Nano roots, whereas it dropped to control value in Baldo at the same time (Figure 5A). The salt-dependent increase of H_2_O_2_ determined a damage of cellular membranes due to lipid peroxidation, as indicated by malondialdehyde (MDA) accumulation. MDA significantly increased during salt-stress exposition in both varieties 24 h after salt treatment (Figure 5B). However, in Vialone Nano, the MDA accumulated to a greater extent in comparison with Baldo (Figure 5B).

**Figure 6:**
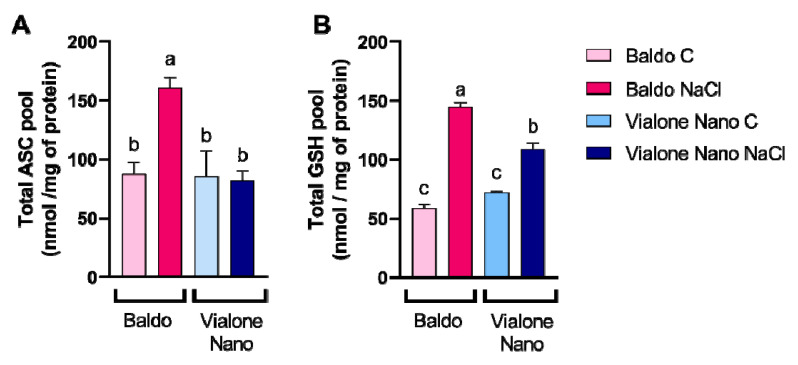
ASC and GSH levels in rice roots under control and salt conditions. (**A**) Ascorbate content in control and treated roots (100 mM NaCl). Changes in total levels of ascorbate (reduced plus oxidized forms; ASC + DHA) pools induced by 100 mM NaCl and determined at 24 h after treatment. (**B**) Glutathione content in control and treated roots (100 mM NaCl). Changes in total levels of glutathione (reduced plus oxidized forms; GSH + GSSG) pools induced by 100 mM NaCl and determined at 24 h after treatment. The reported values are the means of four independent experiments ± standard deviation. Statistical significance was determined by one-way ANOVA followed by a Tukey test (*p* < 0.01). Different letters indicate significant difference.

The overall antioxidant state of control and salt-treated plants showed that the antioxidant capacity increased only in Baldo roots after salt exposure (Figure 5C). Accordingly, Baldo showed higher levels of antioxidant molecules, such as ascorbate and glutathione, in comparison with Vialone Nano 24 h after salt treatment (Figure 6A,B). In particular, no differences in the content of total ascorbate were observed in Vialone Nano between control and treated conditions, while in Baldo roots an increase of approximately 80% was observed. Regarding the total glutathione pool, Baldo roots had a content that was approximately 20% higher than that of Vialone Nano at 24 h after salt treatment. On the other hand, the ascorbate and glutathione redox state was not affected by salt in both the analyzed varieties (data not shown), as well as the activity of the enzymes involved in ROS detoxification and recycling of the oxidized form of ascorbate and glutathione, catalase (CAT), glutathione reductase (GR), ascorbate peroxidase (APX), monodehydroascorbate reductase (MDHAR), and dehydroascorbate reductase (DHAR) (Supplementary Figure S1).

### 3.4. Early Modulation of the Glutathione Metabolism and Sulfur Assimilation in the Tolerant Variety

**Figure 7:**
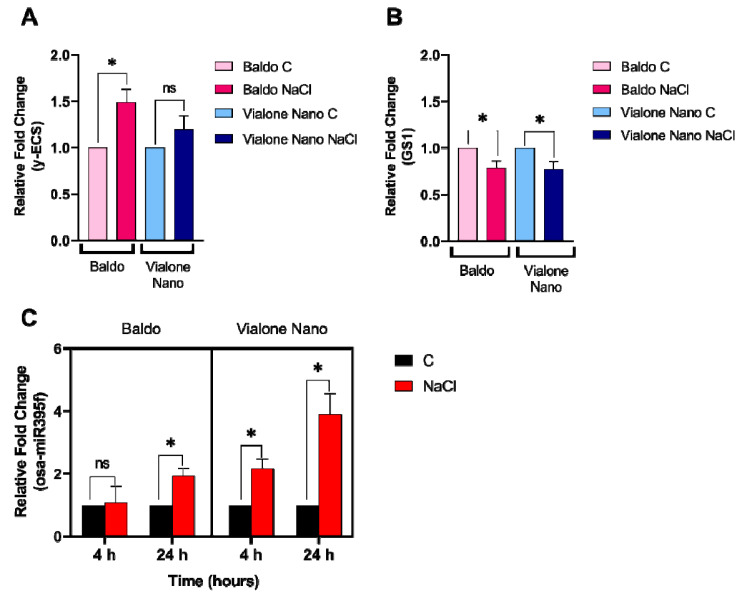
Transcriptional and post-transcriptional regulation of GSH-related genes in rice cultivars dealing with salt stress. Expression profile of genes involved in glutathione biosynthesis, (**A**) γ-glutamylcysteine (γ-EC), and (**B**) glutathione synthetase 1 (GS1) 24 h after salt treatment. The results were normalized for the expression of the housekeeping gene OsACT1. (**C**) Expression profile of miRNA395f gene. Student’s t-test was used to evaluate the significant variations between control and treated condition for each variety. Statistically significant variations are shown (*) using *p*-value ≤  0.01. ns, non-significant. The reported values are the means of four independent experiments ± standard deviation.

The metabolism of glutathione was described in rice to be finely modulated under salt stress in cells as in plants [16,17]. GSH is synthesized from two-consecutive ATP-dependent reactions. In the first reaction, γ-glutamylcysteine (γ-EC) is produced starting from L-glutamate and L-cysteine by γ-glutamylcysteine synthetase (γ-ECS). The second reaction is catalyzed by glutathione synthetase (GS), which adds glycine to C-terminal of γ-EC synthetizing GSH. γ-ECS is described as the major regulatory enzyme in GSH biosynthesis [42]. To better understand the regulation of GSH metabolism in Baldo and Vialone Nano, the expression of the genes coding for the enzymes involved in GSH biosynthesis was analyzed in roots at 24 h after salt treatment (Figure 7A,B). Interestingly, the expression profile of γ-ECS was reported to be different in the two analyzed varieties after salt treatment. Exclusively the tolerant variety Baldo showed a significant increase in the expression of this gene after exposure to salt treatment in accordance with a higher accumulation of total GSH pool observed under salt stress compared to control conditions (Figure 6B and Figure 7A). Moreover, under control conditions, the γ-ECS basal expression was 28% lower in Vialone Nano roots compared to Baldo ones. On the other hand, only a small reduction in the expression of GS was equally observed both in Baldo and in Vialone Nano after treatment (Figure 7B). 

Glutathione levels are also strongly dependent on cysteine availability. Cysteine plays a central role in plant metabolism, since it is a reduced sulfur donor molecule involved in the synthesis of essential metabolites and defense compounds such as thiols and, among these, glutathione. ATP sulfurylase (ATPS) is the first enzyme in the sulfate assimilation pathway [42]. The regulation of the expression profile of this gene and other genes involved in the sulfate assimilation pathway is reported to be finely modulated at a post-transcriptional level by miRNAs belonging to the family of miR395. miRNA are not coding RNA, which regulate gene expression at a post-transcriptional level in a suppressing mode [43,44]. The expression of miR395 is regulated under different abiotic stresses such as during sulfur starvation and drought [45,46,47]. Liang and co-workers demonstrated that miR395 modulates the accumulation of sulfate in rice by directly targeting the transcripts of ATPS and low affinity sulfate transporters [48]. In light of this information, an analysis of the expression of the miR395f was performed. The abundance of miR395f was elevated by the imposed salt-stress treatment in Vialone Nano already after 4 h and in Baldo only at 24 h of treatment (Figure 7C), thus compromising the expression profile of the genes involved in the sulfate assimilation pathway with a different timing.

**Figure 8:**
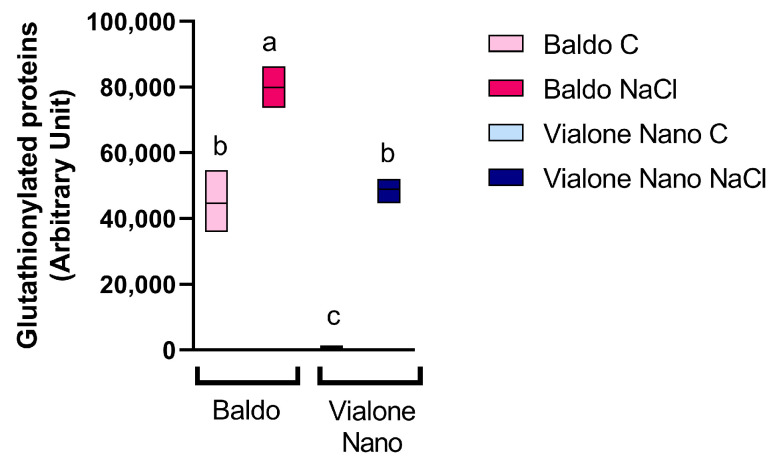
S-Glutathionylation of root proteins in rice seedlings subjected to salt treatment. Densitometric analysis of S-glutathionylation profile of rice proteins extracted from roots at 24 h from salt exposure. The densitometric analysis was performed and quantified by using ImageLab. The density of the blotting signals was normalized to Ponceau S. The reported values are the means of four independent experiments ± standard deviation. Statistical significance was determined by one-way ANOVA followed by a Tukey test (*p* < 0.01). Different letters indicate significant difference.

Glutathione was described to act directly as an antioxidant compound and to protect proteins from oxidation of cysteine residues by disulfide-bond formation through a process called S-glutathionylation [49,50]. The glutathionylation is a reversible redox sensitive post-translational modification involving the formation of a disulfide bond between the glutathione and a cysteine residue on a protein. To better understand the role of glutathione in the defense processes activated in the tolerant variety versus the sensitive one, the profile of glutathionylated proteins was examined in rice roots 24 h after salt treatment (Figure 8).

Both in Baldo and Vialone Nano roots a severe increase in the levels of glutathionylated proteins clearly occurred after salt treatment, thus suggesting the involvement of this post-translational modification in the defense mechanisms activated in rice roots after exposure to salt treatment. 

Interestingly, a marked difference in S-glutathionylated proteins was also observed between Baldo and Vialone Nano under control conditions. In Baldo, the level of S-glutathionylated proteins was much higher in comparison to what was noticed in Vialone Nano roots. Moreover, the glutathionylated protein level observed in Vialone Nano-treated plants was comparable to that of Baldo grown under control conditions (Figure 8).

## 4. Discussion

Increasing crop productivity is a global challenge that requires the identification of new breeding and biotechnology strategies. In the last decades, intraspecies variability has been investigated as an important tool to identify genetic and molecular traits related to plant tolerance to environmental injuries [51]. The identification of species or varieties of agronomic interest able to better tolerate salt stress and of mechanisms allowing such resistance/tolerance is of particular interest for the increasing diffusion of this stress and also because it is a multifactorial stress [4]. 

Root/Shoot (R/S) ratio is considered a parameter correlated with salt tolerance. Indeed, some studies suggested that the capability to maintain a high R/S ratio under salt stress could promote a favorable source/sink ratio thus limiting water deficit and favoring ion exclusion from the shoot [52]. Therefore, the maintenance of root growth is generally related to salt tolerance. Salt stress can affect rice root growth by reducing proliferating activity of meristematic cells [53]. Consistently, the data reported here shows that rice-root cells experienced cell cycle impairment under salt stress. In particular, the percentage of cell nuclei of Vialone Nano root apex in S/G2 phase increased after 1 day of treatment compared to the control. This result suggests a block in cell cycle progression. In Baldo roots, the same salt effect on cell cycle was observed only after 6 days of treatment, and this result is consistent with the delay in root growth inhibition registered in this cultivar under stress in comparison with Vialone Nano (Figure 3). The G2/M checkpoint works to prevent cells presenting DNA damage to enter mitosis [54]. Therefore, an accumulation of cells in G2 phase could indicate a decrease in meristematic activity that finally contributed to affect root growth earlier in Vialone Nano than in Baldo plants (Figure 2D,E). Generally, cell-cycle arrest precedes the activation of cell-death programs [55,56]. Consistently, a marked decrease in cell viability was registered earlier in Vialone Nano roots than in Baldo ones as a consequence of salt stress over the treatment time (Figure 4A).

Consistently with the timing of root-cell death activation, the expression of OsPDCD5, an ortholog to mammalian-programmed cell-death 5, increased in Vialone Nano after 1 day of treatment, whereas in Baldo the same occurred only after 4 days of treatment (Figure 4B). PDCD5 is a protein involved in the activation of apoptosis in mammalian cells, also related to cell-cycle arrest occurring at G2/M checkpoint, as a consequence of DNA damage [57]. The over-expression of the OsPDCD5 gene can induce PCD in transgenic rice [58]. In rice, there is also some evidence suggesting that the regulation of the expression of OsPDCD5 gene is involved in developmental processes, as well as in stress responses. In particular, the down- regulation of the OsPDCD5 gene retarded the PCD process of tapetum cells during pollen development [59], whereas the up-regulation of the OsPDCD5 gene occurred under a low temperature and NaCl treatments [36], and the downregulation of OsPDCD5 increased salt tolerance [60]. Recently, it has also been demonstrated that targeted mutagenesis of OsPDCD5 enhanced grain yield and plant architecture [61].

PCD induction is strictly related to ROS production, depending on the intensity and timing of the accumulation of these compounds [62]. The up-regulation of antioxidant systems has often been reported as part of plant defense responses activated toward environmental stress triggering ROS over-production [63]. Even if it is clearly assessed that the exposure of plants to high soil salinity causes ROS production, a general enhancement of the antioxidant shield was not obviously related to salt tolerance [6,64]. This probably depends on the fact that, as already mentioned, ROS are not only harmful molecules, but also act as key signals [10,65]. ROS timing and intensity are crucial features shaping the role of these compounds [8,66]. 

Since redox signaling is promptly activated when plants perceive a stressing stimulus, ROS and redox parameters have been investigated immediately after stress imposition when the salt-dependent phenotypical effects were not already evident.

Under salt stress, Baldo roots showed a different timing of H_2_O_2_ accumulation compared to Vialone Nano ones. Indeed, in Baldo, H_2_O_2_ production only transiently increased after 4 h from the treatment and dropped to the control value at 24 h after treatment, whereas in Vialone Nano H_2_O_2_ production increased over treatment time (Figure 5A). Consistently, redox damage measured in terms of lipid peroxidation was more evident in Vialone Nano than in Baldo (Figure 5B). 

Baldo and Vialone Nano roots also differed in modulating total antioxidant capability under salt stress, given that Baldo roots are more prone to up-regulate antioxidants than Vialone Nano roots (Figure 5C). Indeed, Baldo exhibited a better capacity to up- regulate both ASC and GSH levels under salt stress than Vialone Nano, which only experienced a GSH increase in the same condition (Figure 6A,B). Consistently, a previous transcriptomic study revealed that the expression of genes involved in GSH metabolism was regulated in response to salt stress in both rice cultivars at root level [17]. Since, the redox state of GSH (data not shown) as well as the activity of the enzymes involved in its redox recycle did not change in the analyzed conditions (Supplementary Figure S1), this study mainly focused on the putative mechanisms controlling GSH biosynthesis. Interestingly, the expression of the enzyme catalyzing the limiting step of GSH production, γ ECS [67], was up-regulated only in Baldo stressed plants (Figure 7A). According to the γ ECS expression under control conditions, Baldo roots showed an innate higher basal GSH biosynthetic capability compared to Vialone Nano ones. Consistently, the amount of glutathionylated proteins was higher in Baldo than in Vialone Nano under both control and treatment conditions (Figure 8). In animals, S-glutathionylation is implicated in the protection of reactive thiolates against irreversible oxidation. The most representative case study of a plant glutathionylated protein is represented by GAPDH, which also seems to protect against oxidative-dependent inactivation by glutathionylation. Indeed, environmental stresses increase protein glutathionylation levels, and, even if the role of this post-translational modification is still to be assessed in plants, this mechanism seems to take part in the redox signaling involved in defense responses [50,68].

Recently, the involvement of miRNA in plant defense responses are the subject of evaluation. In maize, salt-responsive miRNAs have been specifically identified; the expression of these specific miRNAs promptly occurred in a tolerant genotype under salt stress, whereas it lacked or resulted delayed in the sensitive line [69]. 

The regulative feature of miRNAs in determining plant tolerance to stress has been mainly linked to the regulation of redox metabolic and signaling pathways [70]. In maize roots, the expression of miR169q has been suggested to be suppressed by ROS under salt stress. In control conditions, this miRNA negatively controls the expression of the nuclear factor YA8, which plays a role as a transcription factor activating the expression of peroxidase 1. The suppression of miR169 and consequently the expression of YA8 promote salt tolerance [71]. In rice and wheat, miR172a and miR172b have been indicated as positive regulators of salt stress. In particular, miR172a overexpressing lines showed salt tolerance, whereas the downregulation of both miR172a and miR172b were hypersensitive to salt. Indeterminate Spikelet 1 (ISD1), a transcription factor of APETHALA/Ethylene Response Factor (AP/ERF) family, is a target gene of miR172. ISD is a repressor of ROS scavenging genes. Therefore, the up-regulation of miRNA172a/b reduced ISD1 expression, increasing antioxidant shields of cereal crops against salt injury [72]. Recently, in rice, miR528 that has ascorbate oxidase as its target gene has been correlated with salt tolerance. In particular, its expression would preserve the ASC pool, reducing ROS accumulation and consequent oxidative stress [73].

In *Arabidopsis thaliana*, the GSH level seemed to influence the expression of miR395 under S starvation, a condition that induces the up-regulation of this miRNA. Consistently, GSH, being a major antioxidant and thiol compound in the cell, appears to be an S sensor within the cell [74]. In particular, GSH supplementation of S-deficient plants suppressed miR395 induction; on the other hand, in GSH and Thioredoxin (TRX) defective mutants, miR395 expression under S deficiency is partially compromised in a specific mode compared to the expression of other miRNAs [46]. The family of miR365s has been involved in controlling the expression of genes involved in S assimilation. In particular, in dicots as in monocots, miR395 targets the genes coding for ATP sulphurilase (ATPS), enzymes involved in sulfate activation and S transporters’ (SULTR) genes. Thus, miR395 expression affects S assimilation and consequently thiol biosynthesis. In particular, the expression of this miRNA has been suggested to restrict sulfur assimilation in the roots and consequently to promote S transport from root to shoot under S deficiency [75]. Consistently, under biotic stress OsmiR395 expression increases to limit sulfate assimilation and to promote bacterial neutralization by sulfate accumulation [76]. 

It has been suggested that redox signaling promoted by abiotic stress controls miR395 expression [77]. In this study, miR365f expression was responsive to salt treatment in rice roots. Interestingly, the level of this miRNA increased with a different timing in sensitive and tolerant cultivars (Figure 7B). The early up-regulation of miR365f in Vialone Nano roots might affect thiol biosynthesis consistently, with the more moderate increase of GSH registered in Vialone Nano stressed plants in comparison with Baldo ones. 

## 5. Conclusions

**Figure 9:**
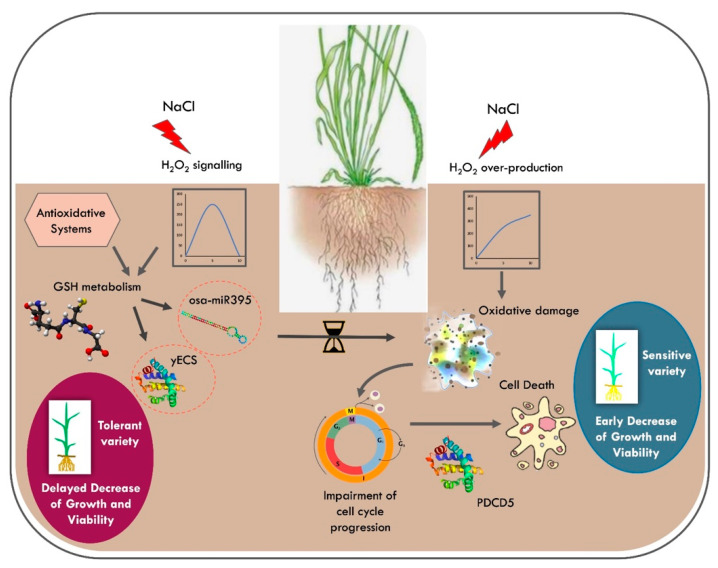
Redox strategy underpinning salt tolerance in rice. A differential modulation of the redox balance is pivotal for the protection against salt stress in the tolerant rice variety. In Baldo, a rapid and effective modulation of the antioxidative systems allows a delay of the oxidative damage to the cellular components and consequent impairment of cell-cycle progression and cell viability due to a shift toward an ROS signaling mode instead of a damaging one. In this context, glutathione metabolism was identified as a possible pathway, which contributes to conferring salt tolerance on rice. A variety-specific modulation of the expression of the enzyme γ ECS and regulation, at a post-transcriptional level, of sulfur assimilation, mediated by osa-miR395, can contribute to the delayed decrease in plant growth and viability observed in tolerant Baldo rice variety.

In conclusion, the results reported in this study support the idea that the activation of a complex redox strategy is more effective in improving plant capability to cope with environmental injury than a general enhancement of antioxidant systems. Indeed, ROS toxic nature depends on the aptitude of these compounds to oxide cell components, which, consequently, activate an oxidative cascade that amplifies ROS dependent damages. In this framework, antioxidants are pivotal in plant protection against stress since they, reducing ROS accumulation, do not allow the amplification of ROS toxic effects and promote the switch of these compounds toward their signaling mode. However, the timing of the modulation of antioxidant systems is crucial in determining plant fate (Figure 9; Refs. [9,66]). Consistently, as a consequence of an efficient tuning of ROS levels, suffering symptoms, including those related to growth impairment, lately appeared and were less pronounced in the tolerant cultivar compared to the sensitive one (Figure 1 and Figure 2). The activation of cell death was also retarded in Baldo roots compared to Vialone Nano ones (Figure 4). The innate ability of Baldo to cope with salt stress would thus depend on the capability of this cultivar to promptly restrain H_2_O_2_ levels by up-regulating antioxidant levels after the imposition of stress (Figure 5C and Figure 6). Consequently, Baldo roots experienced only a transient increase of H_2_O_2_, which shows a trend typical of a redox signal able to activate defense responses instead of promoting general damage (Figure 5A; [10,17]). Interestingly, the increase of GSH levels, in terms of free peptide and GSH bounded to proteins, involved transcriptional and post-transcriptional regulation only in Baldo (Figure 7A,C). Therefore, the multifactorial nature of GSH metabolism regulation could be determinant in conferring salt tolerance and, thus, this feature should attract further investigation.

## Data Availability

Data is contained within the article.

## Supplementary Materials

The following supporting information can be downloaded at: https://www.mdpi.com/article/10.3390/antiox11061114/s1, Figure S1: Changes in the activity of redox-related enzymes; Table S1: Primer sequences for Real Time analyses.

## References

[1] Zaki Mostafa Ali F. (2011). The Determinants of Salinity Tolerance in Maize (Zea Mays L.).

[2] Safdar H., Amin A., Shafiq Y., Ali A., Yasin R., Sarwar M.I. (2019). Abbas Shoukat, Maqsood Ul Hussan, Muhammad Ishtiaq Sarwar. A Review: Impact of Salinity on Plant Growth. Nat. Sci..

[3] Hasegawa P.M., Bressan R.A., Zhu J.-K., Bohnert H.J. (2000). Plant Cellular and Molecular Responses to High Salinity. Annu. Rev. Plant Physiol. Plant Mol. Biol..

[4] Yang Y., Guo Y. (2018). Elucidating the Molecular Mechanisms Mediating Plant Salt-Stress Responses. New Phytol..

[5] Hsiao T.C., Xu L. (2000). Sensitivity of Growth of Roots versus Leaves to Water Stress: Biophysical Analysis and Relation to Water Transport. J. Exp. Bot..

[6] Hossain M.S., Dietz K.-J. (2016). Tuning of Redox Regulatory Mechanisms, Reactive Oxygen Species and Redox Homeostasis under Salinity Stress. Front. Plant Sci..

[7] Demidchik V., Cuin T., Svistunenko D., Smith S., Miller A., Shabala S., Sokolik A., Yurin V. (2010). Arabidopsis Root K+-Efflux Conductance Activated by Hydroxyl Radicals: Single-Channel Properties, Genetic Basis and Involvement in Stress-Induced Cell Death. J. Cell Sci..

[8] Locato V., Gadaleta C., De Gara L., De Pinto M.C. (2008). Production of Reactive Species and Modulation of Antioxidant Network in Response to Heat Shock: A Critical Balance for Cell Fate. Plant Cell Environ..

[9] Locato V., Paradiso A., Sabetta W., De Gara L., de Pinto M.C., Wendehenne D. (2016). Chapter Nine-Nitric Oxide and Reactive Oxygen Species in PCD Signaling. Advances in Botanical Research.

[10] Considine M.J., Foyer C.H. (2021). Stress Effects on the Reactive Oxygen Species-Dependent Regulation of Plant Growth and Development. J. Exp. Bot..

[11] Zagorchev L., Seal C.E., Kranner I., Odjakova M. (2013). A Central Role for Thiols in Plant Tolerance to Abiotic Stress. Int. J. Mol. Sci..

[12] Fatma M., Asgher M., Masood A., Khan N.A. (2014). Excess Sulfur Supplementation Improves Photosynthesis and Growth in Mustard under Salt Stress through Increased Production of Glutathione. 2014, 107, 55–63. Environ. Exp. Bot..

[13] Fatma M., Masood A., Per T.S., Khan N.A. (2016). Nitric Oxide Alleviates Salt Stress Inhibited Photosynthetic Performance by Interacting with Sulfur Assimilation in Mustard. Front. Plant Sci..

[14] Kaur N., Dhawan M., Sharma I., Pati P.K. (2016). Interdependency of Reactive Oxygen Species Generating and Scavenging System in Salt Sensitive and Salt Tolerant Cultivars of Rice. BMC Plant Biol..

[15] Bertazzini M., Sacchi G.A., Forlani G. (2018). A Differential Tolerance to Mild Salt Stress Conditions among Six Italian Rice Genotypes Does Not Rely on Na^+^ Exclusion from Shoots. J. Plant Physiol..

[16] Formentin E., Sudiro C., Ronci M.B., Locato V., Barizza E., Stevanato P., Ijaz B., Zottini M., De Gara L., Lo Schiavo F. (2018). H_2_O_2_ Signature and Innate Antioxidative Profile Make the Difference Between Sensitivity and Tolerance to Salt in Rice Cells. Front. Plant Sci..

[17] Formentin E., Sudiro C., Perin G., Riccadonna S., Barizza E., Baldoni E., Lavezzo E., Stevanato P., Sacchi G.A., Fontana P. (2018). Transcriptome and Cell Physiological Analyses in Different Rice Cultivars Provide New Insights Into Adaptive and Salinity Stress Responses. Front. Plant Sci..

[18] Hoagland D.R., Arnon D.I. (1950). The Water-Culture Method for Growing Plants without Soil. Circ. Calif. Agric. Exp. Stn..

[19] Counce P.A., Keisling T.C., Mitchell A.J. (2000). A Uniform, Objective, and Adaptive System for Expressing Rice Development. Crop Sci..

[20] Willis O.O., Mouti M.E., Sila D.N., Mwasaru M., Thiongo G., Murage H., Ojijo N.O. (2013). Physico-Chemical Properties and Antioxidant Potential of Syrup Prepared from ‘Madhura’ Sweet Sorghum (*Sorghum Bicolor* L. Moench) Cultivar Grown at Different Locations in Kenya. Sugar Tech..

[21] Ferrer M.A., Cimini S., López-Orenes A., Calderón A.A., De Gara L. (2018). Differential Pb Tolerance in Metallicolous and Non-Metallicolous Zygophyllum Fabago Populations Involves the Strengthening of the Antioxidative Pathways. Environ. Exp. Bot..

[22] Romero-Puertas M.C., Rodríguez-Serrano M., Corpas F.J., Gómez M., Del Río L.A., Sandalio L.M. (2004). Cadmium-Induced Subcellular Accumulation of O_2_^−^ and H_2_O_2_ in Pea Leaves. Plant Cell Environ..

[23] Varkonyi-Gasic E., Wu R., Wood M., Walton E.F., Hellens R.P. (2007). Protocol: A Highly Sensitive RT-PCR Method for Detection and Quantification of MicroRNAs. Plant Methods.

[24] Livak K.J., Schmittgen T.D. (2001). Analysis of Relative Gene Expression Data Using Real-Time Quantitative PCR and the 2(-Delta Delta C(T)) Method. Methods.

[25] Schmittgen T.D., Livak K.J. (2008). Analyzing Real-Time PCR Data by the Comparative C(T) Method. Nat. Protoc..

[26] Zhao L., Wang P., Hou H., Zhang H., Wang Y., Yan S., Huang Y., Li H., Tan J., Hu A. (2014). Transcriptional Regulation of Cell Cycle Genes in Response to Abiotic Stresses Correlates with Dynamic Changes in Histone Modifications in Maize. PLoS ONE.

[27] Sabetta W., Vandelle E., Locato V., Costa A., Cimini S., Bittencourt Moura A., Luoni L., Graf A., Viggiano L., De Gara L. (2019). Genetic Buffering of Cyclic AMP in *Arabidopsis Thaliana* Compromises the Plant Immune Response Triggered by an Avirulent Strain of Pseudomonas Syringae Pv. Tomato. Plant J..

[28] De Pinto M.C., Francis D., De Gara L. (1999). The Redox State of the Ascorbate-Dehydroascorbate Pair as a Specific Sensor of Cell Division in Tobacco BY-2 Cells. Protoplasma.

[29] García-Ulloa A., Sanjurjo L., Cimini S., Encina A., Martínez-Rubio R., Bouza R., Barral L., Estévez-Pérez G., Novo-Uzal E., De Gara L. (2020). Overexpression of ZePrx in Nicotiana Tabacum Affects Lignin Biosynthesis without Altering Redox Homeostasis. Front. Plant Sci..

[30] Hodges D.M., DeLong J.M., Forney C.F., Prange R.K. (1999). Improving the Thiobarbituric Acid-Reactive-Substances Assay for Estimating Lipid Peroxidation in Plant Tissues Containing Anthocyanin and Other Interfering Compounds. Planta.

[31] Pasqualetti V., Locato V., Fanali C., Mulinacci N., Cimini S., Morgia A.M., Pasqua G., De Gara L. (2021). Comparison between In Vitro Chemical and Ex Vivo Biological Assays to Evaluate Antioxidant Capacity of Botanical Extracts. Antioxidants.

[32] Locato V., Uzal E., Cimini S., Zonno M., Evidente A., Micera A., Foyer C., De Gara L. (2015). Low Concentrations of the Toxin Ophiobolin A Lead to an Arrest of the Cell Cycle and Alter the Intracellular Partitioning of Glutathione between the Nuclei and Cytoplasm. J. Exp. Bot..

[33] Pushpam R., Manonmani S., Varthini N.V., Robin S. (2018). Studies on Yield, Root Characters Related to Drought Tolerance and Their Association in Upland Rice Genotypes. Electron. J. Plant Breed..

[34] Xu N., Chu Y., Chen H., Li X., Wu Q., Jin L., Wang G., Huang J. (2018). Rice Transcription Factor OsMADS25 Modulates Root Growth and Confers Salinity Tolerance via the ABA–Mediated Regulatory Pathway and ROS Scavenging. PLOS Genet..

[35] Seo D.H., Seomun S., Choi Y.D., Jang G. (2020). Root Development and Stress Tolerance in Rice: The Key to Improving Stress Tolerance without Yield Penalties. Int. J. Mol. Sci..

[36] Su W., Wu J., Wei C., Li K., He G., Attla K., Qian X., Yang J. (2006). Interaction between Programmed Cell Death 5 and Calcineurin B-like Interacting Protein Kinase 23 in *Oryza Sativa*. Plant Sci..

[37] Sun F., Qi W., Qian X., Wang Q., Yang M., Dong X., Yang J. (2012). Investigating the Role of OsPDCD5, a Homolog of the Mammalian PDCD5, in Programmed Cell Death by Inducible Expression in Rice. Plant Mol. Biol. Report..

[38] Falcone Ferreyra M.L., Casadevall R., D’Andrea L., AbdElgawad H., Beemster G.T.S., Casati P. (2016). AtPDCD5 Plays a Role in Programmed Cell Death after UV-B Exposure in Arabidopsis. Plant Physiol..

[39] De Pinto M.C., Locato V., Paradiso A., De Gara L. (2015). Role of Redox Homeostasis in Thermo-Tolerance under a Climate Change Scenario. Ann. Bot..

[40] Mittler R. (2017). ROS Are Good. Trends Plant Sci..

[41] Farnese F.S., Menezes-Silva P.E., Gusman G.S., Oliveira J.A. (2016). When Bad Guys Become Good Ones: The Key Role of Reactive Oxygen Species and Nitric Oxide in the Plant Responses to Abiotic Stress. Front. Plant Sci..

[42] Noctor G., Mhamdi A., Chaouch S., Han Y., Neukermans J., Marquez-Garcia B., Queval G., Foyer C.H. (2012). Glutathione in Plants: An Integrated Overview. Plant Cell Environ..

[43] Bartel D.P. (2004). MicroRNAs: Genomics, Biogenesis, Mechanism, and Function. Cell.

[44] Reinhart B.J., Weinstein E.G., Rhoades M.W., Bartel B., Bartel D.P. (2002). MicroRNAs in Plants. Genes Dev..

[45] Jones-Rhoades M.W., Bartel D.P. (2004). Computational Identification of Plant MicroRNAs and Their Targets, Including a Stress-Induced MiRNA. Mol. Cell.

[46] Jagadeeswaran G., Li Y.-F., Sunkar R. (2014). Redox Signaling Mediates the Expression of a Sulfate-Deprivation-Inducible MicroRNA395 in Arabidopsis. Plant J..

[47] Cao J., Gulyás Z., Kalapos B., Boldizsár Á., Liu X., Pál M., Yao Y., Galiba G., Kocsy G. (2019). Identification of a Redox-Dependent Regulatory Network of MiRNAs and Their Targets in Wheat. J. Exp. Bot..

[48] Liang G., Yang F., Yu D. (2010). MicroRNA395 Mediates Regulation of Sulfate Accumulation and Allocation in *Arabidopsis Thaliana*. Plant J..

[49] Dixon D.P., Skipsey M., Grundy N.M., Edwards R. (2005). Stress-Induced Protein S-Glutathionylation in Arabidopsis. Plant Physiol..

[50] Zaffagnini M., Bedhomme M., Lemaire S.D., Trost P. (2012). The Emerging Roles of Protein Glutathionylation in Chloroplasts. Plant Sci..

[51] Voesenek L., Bailey-Serres J. (2009). Plant Biology: Genetics of High-Rise Rice. Nature.

[52] Acosta-Motos J.R., Ortuño M.F., Bernal-Vicente A., Diaz-Vivancos P., Sanchez-Blanco M.J., Hernandez J.A. (2017). Plant Responses to Salt Stress: Adaptive Mechanisms. Agronomy.

[53] Huang Y., Zhou J., Li Y., Quan R., Wang J., Huang R., Qin H. (2021). Salt Stress Promotes Abscisic Acid Accumulation to Affect Cell Proliferation and Expansion of Primary Roots in Rice. Int. J. Mol. Sci..

[54] Zhou B.-B.S., Elledge S.J. (2000). The DNA Damage Response: Putting Checkpoints in Perspective. Nature.

[55] Kadota Y., Watanabe T., Fujii S., Higashi K., Sano T., Nagata T., Hasezawa S., Kuchitsu K. (2004). Crosstalk between Elicitor-Induced Cell Death and Cell Cycle Regulation in Tobacco BY-2 Cells. Plant J..

[56] Zebell S.G., Dong X. (2015). Cell Cycle Regulators and Cell Death in Immunity. Cell Host Microbe.

[57] Li P., Fei H., Wang L., Xu H., Zhang H., Zheng L. (2018). PDCD5 Regulates Cell Proliferation, Cell Cycle Progression and Apoptosis. Oncol. Lett..

[58] Attia K., Li K.-G., Wei C., He G.-M., Su W., Yang J.-S. (2005). Overexpression of the OsPDCD5 Gene Induces Programmed Cell Death in Rice. J. Integr. Plant Biol..

[59] Wang Y., Attia K., Zha X., Abdelkhalik A., Zhang S., Qian X., Dong X., Sun F., Yang J., Lightfoot D. (2010). Down-Regulation of the OsPDCD5 Gene Induced Photoperiod-Sensitive Male Sterility in Rice. Nat. Preced..

[60] Yang M., Sun F., Wang S., Qi W., Wang Q., Dong X., Yang J., Luo X. (2013). Down-Regulation of OsPDCD5, a Homolog of the Mammalian PDCD5, Increases Rice Tolerance to Salt Stress. Mol. Breed..

[61] Dong S., Dong X., Han X., Zhang F., Zhu Y., Xin X., Wang Y., Hu Y., Yuan D., Wang J. (2021). OsPDCD5 Negatively Regulates Plant Architecture and Grain Yield in Rice. Proc. Natl. Acad. Sci. USA.

[62] De Pinto M.C., Locato V., De Gara L. (2012). Redox Regulation in Plant Programmed Cell Death. Plant Cell Environ..

[63] Locato V., Cimini S., De Gara L. (2018). ROS and Redox Balance as Multifaceted Players of Cross-Tolerance: Epigenetic and Retrograde Control of Gene Expression. J. Exp. Bot..

[64] Pilarska M., Wiciarz M., Jajić I., Kozieradzka-Kiszkurno M., Dobrev P., Vanková R., Niewiadomska E. (2016). A Different Pattern of Production and Scavenging of Reactive Oxygen Species in Halophytic Eutrema Salsugineum (Thellungiella Salsuginea) Plants in Comparison to *Arabidopsis Thaliana* and Its Relation to Salt Stress Signaling. Front. Plant Sci..

[65] Baxter A., Mittler R., Suzuki N. (2014). ROS as Key Players in Plant Stress Signalling. J. Exp. Bot..

[66] Ijaz B., Formentin E., Ronci B., Locato V., Barizza E., Hyder M.Z., Schiavo F.L., Yasmin T. (2019). Salt Tolerance in Indica Rice Cell Cultures Depends on a Fine Tuning of ROS Signalling and Homeostasis. PLoS ONE.

[67] Hasanuzzaman M., Nahar K., Anee T.I., Fujita M. (2017). Glutathione in Plants: Biosynthesis and Physiological Role in Environmental Stress Tolerance. Physiol. Mol. Biol. Plants.

[68] Dorion S., Ouellet J.C., Rivoal J. (2021). Glutathione Metabolism in Plants under Stress: Beyond Reactive Oxygen Species Detoxification. Metabolites.

[69] Ding D., Zhang L., Wang H., Liu Z., Zhang Z., Zheng Y. (2009). Differential Expression of MiRNAs in Response to Salt Stress in Maize Roots. Ann. Bot..

[70] Cimini S., Gualtieri C., Macovei A., Balestrazzi A., De Gara L., Locato V. (2019). Redox Balance-DDR-MiRNA Triangle: Relevance in Genome Stability and Stress Responses in Plants. Front. Plant Sci..

[71] Xing L., Zhu M., Luan M., Zhang M., Jin L., Liu Y., Zou J., Wang L., Xu M. (2022). MiR169q and NUCLEAR FACTOR YA8 Enhance Salt Tolerance by Activating PEROXIDASE1 Expression in Response to ROS. Plant Physiol..

[72] Cheng X., He Q., Tang S., Wang H., Zhang X., Lv M., Liu H., Gao Q., Zhou Y., Wang Q. (2021). The MiR172/IDS1 Signaling Module Confers Salt Tolerance through Maintaining ROS Homeostasis in Cereal Crops. New Phytol..

[73] Wang M., Guo W., Li J., Pan X., Pan L., Zhao J., Zhang Y., Cai S., Huang X., Wang A. (2021). The MiR528-AO Module Confers Enhanced Salt Tolerance in Rice by Modulating the Ascorbic Acid and Abscisic Acid Metabolism and ROS Scavenging. J. Agric. Food Chem..

[74] Takahashi H., Yamazaki M., Sasakura N., Watanabe A., Leustek T., Engler J.d.A., Engler G., Van Montagu M., Saito K. (1997). Regulation of Sulfur Assimilation in Higher Plants: A Sulfate Transporter Induced in Sulfate-Starved Roots Plays a Central Role in *Arabidopsis Thaliana*. Proc. Natl. Acad. Sci. USA.

[75] Matthewman C.A., Kawashima C.G., Húska D., Csorba T., Dalmay T., Kopriva S. (2012). MiR395 Is a General Component of the Sulfate Assimilation Regulatory Network in Arabidopsis. FEBS Lett..

[76] Yang Z., Hui S., Lv Y., Zhang M., Chen D., Tian J., Zhang H., Liu H., Cao J., Xie W. (2022). MiR395-Regulated Sulfate Metabolism Exploits Pathogen Sensitivity to Sulfate to Boost Immunity in Rice. Mol. Plant.

[77] Panda S.K., Sunkar R. (2015). Nutrient- and Other Stress-Responsive MicroRNAs in Plants: Role for Thiol-Based Redox Signaling. Plant Signal. Behav..

