# Supplementary material for: A Multifactorial Regulation of Glutathione Metabolism behind Salt Tolerance in Rice
Source: Antioxidants (Basel). 2022 Jun 3;11(6):1114. doi: 10.3390/antiox11061114 (PMC9219684; doi:10.3390/antiox11061114)
Supplement: Supplementary file 1 [file antioxidants-11-01114-s001.zip › antioxidants-1760982-supplementary.pdf]

Supplementary Data

Table S1. Primer sequences for Real Time analyses.

Gene Name	Locus ID	Primer Name	Primer Sequence
**Ubiquitin**	Os01g0328400	OsUBQ5-FW	TTCTACAAGGTGGACGACGC
		OsUBQ5-Rev	AGATCAGAGCAAAGCGAGCA
**Actin**	Os03g0718100	OsAct1-FW	TGGCATCTCTCAGCACATTCC
		OsAct1-Rev	TGCACAATGGATGGGCCAGA
**PDCD5 programmed cell death 5**	Os05g0547850	OsPDCD5-FW	ATCCGCCCCTGTTGTATGTG
		OsPDCD5-Rev	ATGAAGCAAGGAGCCAACCA
**GS1**	Os11g0642800	OsGS1-FW	GTGGCGTTTGCTTTGAGGAC
		OsGS1-Rev	GTTCTGAGCTTCCAAAGGGTT
**yECS**	Os05g0129000	OsECS-FW	AGCCTTTGTAGGTGGTGTCTTTTA
		OsECS-Rev	AAGAGGGTCTGGTACAAACAGTG
**miRNA395f**	Chr4: 31804774-31804855 MI0001043	OsmiR395f-UPL-RT	GTTGGCTCTGGTGCAGGGTCCGAGGTATTCGCACCAGAGCCAACGAGTTC
		OsmiR395f-FW	GGCGGGTGAATTGTTTGGG
		Rev Universal	GTGCAGGGTCCGAGGT

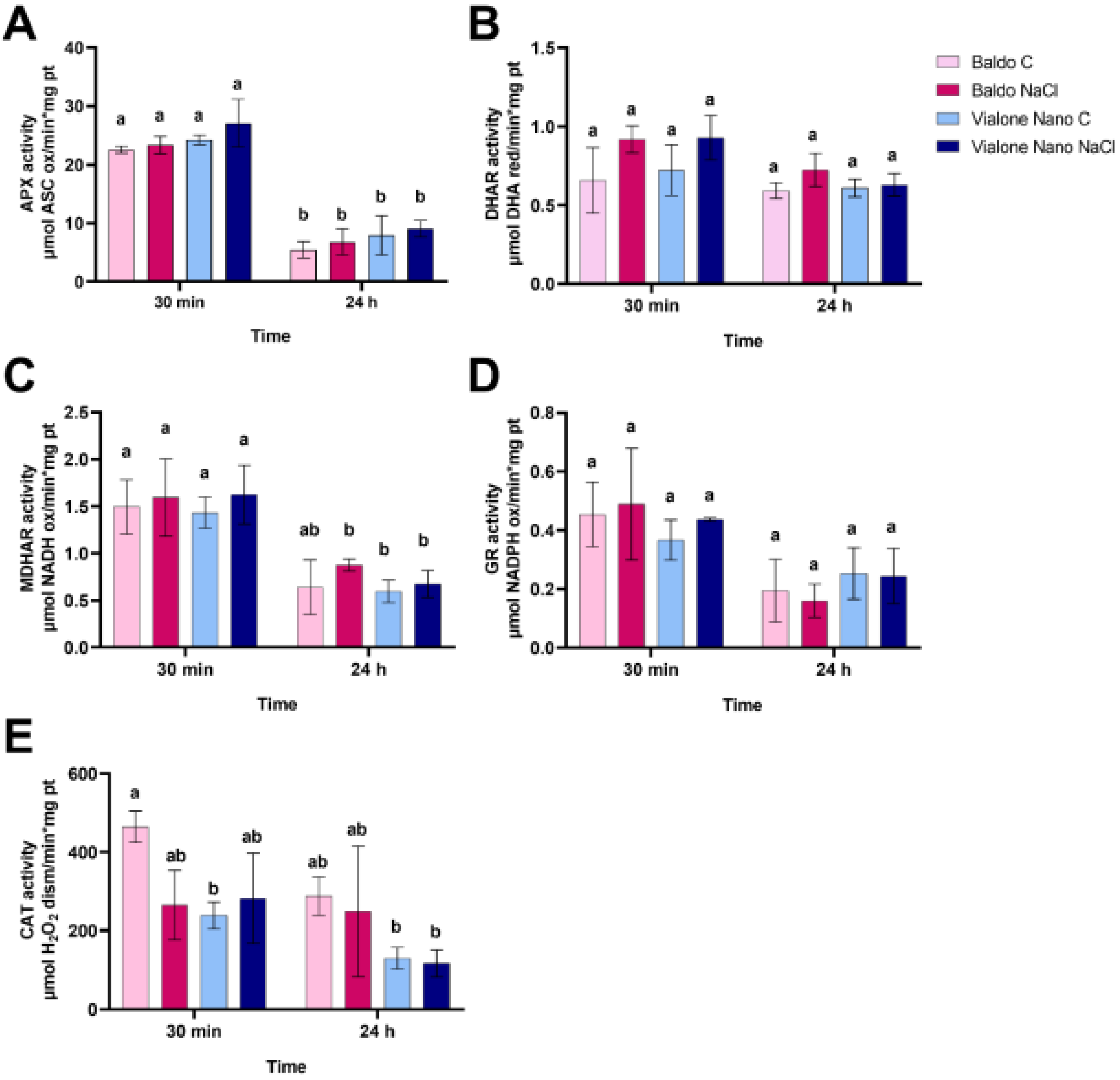

Figure S1. Changes in the activity of redox-related enzymes. Ascorbate peroxidase (APX); dehydroascorbate reductase (DHAR); monodehydroascorbate reductase (MDHAR); glutathione reductase (GR); catalase (CAT). The reported values are the means of four independent experiments ± standard deviation. Statistical significance was determined by one-way ANOVA followed by a Tukey test ($P < 0.05$). Different letters indicate significant difference.
